# (*E*)-7-(4-Chloro­phen­yl)-5,7-dihydro-4*H*-pyrano[3,4-*c*]isoxazole-3-carbaldehyde oxime

**DOI:** 10.1107/S1600536811005575

**Published:** 2011-02-19

**Authors:** Hyun Sub Lim, Hyung Jin Kim, Enkhzul Otgonbaatar, Chee-Hun Kwak

**Affiliations:** a School of Applied Chemical Engineering, Chonnam National University, Gwangju 500-757, Republic of Korea; b Department of Chemistry, Sunchon National University, Sunchon 540-742, Republic of Korea

## Abstract

In the title compound, C_13_H_11_ClN_2_O_3_, the nine-membered bicycle includes an oxime group having the C=N group in an *E* configuration. The isoxazole ring is almost planar [r.m.s. deviation = 0.0056 Å]; the dihedral angle between the isoxazole and 4-chloro­phenyl ring is 75.60 (5)°. In the crystal, inter­molecular O—H⋯N_isoxazole_ hydrogen bonds give rise to chains running along the *b* axis.

## Related literature

For the synthesis, see: Kim & Lee (1994 ▶).
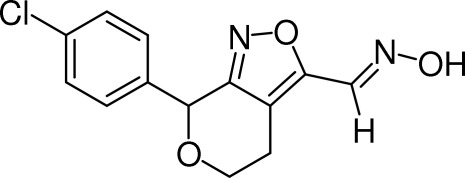


## Experimental

### 

#### Crystal data

C_13_H_11_ClN_2_O_3_
*M* *_r_* = 278.69
Monoclinic, 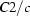

*a* = 32.748 (2) Å
*b* = 8.8501 (5) Å
*c* = 8.6366 (5) Å
β = 90.478 (2)°
*V* = 2503.0 (3) Å^3^
*Z* = 8
Mo *K*α radiation
μ = 0.31 mm^−1^
*T* = 293 K
0.8 × 0.6 × 0.4 mm

#### Data collection

Rigaku R-AXIS RAPID II-S diffractometer
Absorption correction: multi-scan (*RAPID-AUTO*; Rigaku, 2008 ▶) *T*_min_ = 0.800, *T*_max_ = 0.833
11943 measured reflections
2860 independent reflections
2467 reflections with *I* > 2σ(*I*)
*R*_int_ = 0.050

#### Refinement

*R*[*F* ^2^ > 2σ(*F* ^2^)] = 0.038
*wR*(*F* ^2^) = 0.101
*S* = 1.04
2860 reflections
172 parameters
H-atom parameters constrained
Δρ_max_ = 0.38 e Å^−3^
Δρ_min_ = −0.25 e Å^−3^

### 

Data collection: *RAPID-AUTO* (Rigaku, 2008 ▶); cell refinement: *RAPID-AUTO*; data reduction: *RAPID-AUTO*; program(s) used to solve structure: *SHELXS97* (Sheldrick, 2008 ▶); program(s) used to refine structure: *SHELXL97* (Sheldrick, 2008 ▶); molecular graphics: *ORTEP-3* (Farrugia, 1997 ▶); software used to prepare material for publication: *SHELXL97*.

## Supplementary Material

Crystal structure: contains datablocks I, global. DOI: 10.1107/S1600536811005575/ng5111sup1.cif

Structure factors: contains datablocks I. DOI: 10.1107/S1600536811005575/ng5111Isup2.hkl

Additional supplementary materials:  crystallographic information; 3D view; checkCIF report

## Figures and Tables

**Table 1:** Hydrogen-bond geometry (Å, °)

*D*—H⋯*A*	*D*—H	H⋯*A*	*D*⋯*A*	*D*—H⋯*A*
O3—H3⋯N1^i^	0.82	2.07	2.7920 (15)	147

Symmetry code: (i) 
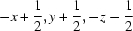
.

**Fig. 1.**
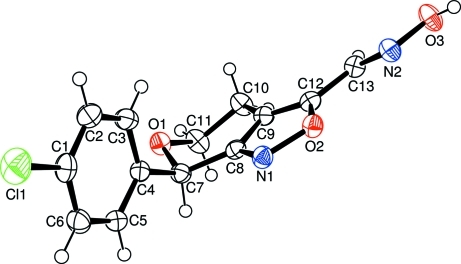
The structure of the title compound with displacement ellipsoids drawn at the 50% probability level for non-H atoms.

**Fig. 2.**
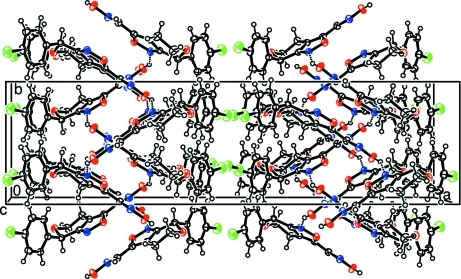
A packing diagram of the title compound. Dashed bonds represent hydrogen bonds.

## supplementary crystallographic information

### Comment

Fused bicyclic isoxazoles, such as dihydrofuro- and dihydropyrano[3,4-*c*]isoxazole,particularly have fungicidal activities against some plant pathogens. To find a new lead compound as plant fungicide and to study the structure-activity relationship (SAR), we are interested in the solid-state structures of the fused bicyclic isoxazoles.The title compound, C_13_H_11_ClN_2_O_3_, forms bicycle adjointed 5-membered isoxazole ring and 6-membered oxane ring in C8 and C9 position and it adopts *E* conformation (Fig.1). Isoxazole ring is a plane with the largest deviation of 0.0081 (8)Å from the least suare plane.The C7 and C10 atoms lie in the isoxazole ring plane with the largest deviation of 0.00157 (7)Å (C7) from the least-squares plane of the isoxazole ring. The dihedral angle between isoxazole and 4-chlorophenyl ring is 75.60 (5)°.The compound displays intermolecular hydrogen boning between oxyen(O3) of oxime and nitrogen(symmetric code:O3 and H3 = x,y,z+1,N3 = -x+1/2, y+1/2, -z+/2)isoxazole with a distance between O3 and N1 of 2.79 (2)Å )(Fig. 2 and Table 1). This intermolecular hydrogen bonding forms 1-D zig-zag chain of of titled compound in crystalline solid.

### Experimental

A mixture of 7-(4-chlorophenyl)-5,7-dihydro-4*H*-pyrano[3,4-*c*]isoxazole-3-carbaldehyde (1.50 g, 5.69 mmol), HONH_2_.HCl (593 mg, 8.53 mmol) and NaOAc (700 mg, 8.53 mmol) in EtOH (30 ml) was stirred for 3 h at 25 °C. After filtration of the reaction mixture, the filtrate was concentrated under vacuum to give crude product, which was chromatographed on SiO_2_ eluting with *n*-hexane/EtOAc (2:1) solution to afford an isomeric mixture mixture (*E*:*Z* = 9:2). Single crystals of the (*E*)-isomer suitable for X-ray analysis were obtained by slow evaporation from an *n*-hexane/EtOAc solution at room temperature.

### Refinement

H atoms were positioned geometrically and allowed to ride on their respective parent atoms [C—H = 0.93 (CH, *sp*^2^), 0.98 (CH, *sp*^3^) or 0.97 Å (CH_2_) and *O*—*H*(hydroxyl) =0.82, and *U*_iso_(H) = 1.2*U*_eq_(C),*U*_iso_(H) = 1.2*U*_eq_(O)].

### Crystal data

**Table:**

C_13_H_11_ClN_2_O_3_	Z = 8
*M**_r_* = 278.69	*D*_x_ = 1.479 Mg m^−^^3^
Monoclinic, *C*2/*c*	Mo *K*α radiation, λ = 0.71073 Å
*a* = 32.748 (2) Å	Cell parameters from 12348 reflections
*b* = 8.8501 (5) Å	θ = 27.5–3.4°
*c* = 8.6366 (5) Å	µ = 0.31 mm^−^^1^
β = 90.478 (2)°	*T* = 293 K
*V* = 2503.0 (3) Å^3^	Block, colorless
*Z* = 8	0.8 × 0.6 × 0.4 mm
*F*(000) = 1152	

### Data collection

**Table:**

Rigaku R-AXIS RAPID II-S diffractometer	2860 independent reflections
Radiation source: fine-focus sealed tube	2467 reflections with *I* > 2σ(*I*)
graphite	*R*_int_ = 0.050
ω scans	θ_max_ = 27.5°, θ_min_ = 3.4°
Absorption correction: multi-scan (*RAPID-AUTO*; Rigaku, 2008)	*h* = −42→42
*T*_min_ = 0.800, *T*_max_ = 0.833	*k* = −10→11
11943 measured reflections	*l* = −11→11

### Refinement

**Table:**

Refinement on *F*^2^	Primary atom site location: structure-invariant direct methods
Least-squares matrix: full	Secondary atom site location: difference Fourier map
*R*[*F*^2^ > 2σ(*F*^2^)] = 0.038	Hydrogen site location: inferred from neighbouring sites
*wR*(*F*^2^) = 0.101	H-atom parameters constrained
*S* = 1.04	*w* = 1/[σ^2^(*F*_o_^2^) + (0.0482*P*)^2^ + 1.5098*P*] where *P* = (*F*_o_^2^ + 2*F*_c_^2^)/3
2860 reflections	(Δ/σ)_max_ < 0.001
172 parameters	Δρ_max_ = 0.38 e Å^−^^3^
0 restraints	Δρ_min_ = −0.25 e Å^−^^3^

### Special details

**Table:**

Geometry. All e.s.d.'s (except the e.s.d. in the dihedral angle between two l.s. planes) are estimated using the full covariance matrix. The cell e.s.d.'s are taken into account individually in the estimation of e.s.d.'s in distances, angles and torsion angles; correlations between e.s.d.'s in cell parameters are only used when they are defined by crystal symmetry. An approximate (isotropic) treatment of cell e.s.d.'s is used for estimating e.s.d.'s involving l.s. planes.
Refinement. Refinement of *F*^2^ against ALL reflections. The weighted *R*-factor *wR* and goodness of fit *S* are based on *F*^2^, conventional *R*-factors *R* are based on *F*, with *F* set to zero for negative *F*^2^. The threshold expression of *F*^2^ > σ(*F*^2^) is used only for calculating *R*-factors(gt) *etc*. and is not relevant to the choice of reflections for refinement. *R*-factors based on *F*^2^ are statistically about twice as large as those based on *F*, and *R*- factors based on ALL data will be even larger.

### Fractional atomic coordinates and isotropic or equivalent isotropic displacement parameters (Å^2^)

**Table:**

	*x*	*y*	*z*	*U*_iso_*/*U*_eq_	
Cl1	0.011720 (14)	0.77709 (6)	0.05872 (5)	0.05107 (15)	
O3	0.30644 (3)	1.11893 (12)	−0.46915 (11)	0.0345 (2)	
H3	0.3206	1.1478	−0.3961	0.052*	
O2	0.21571 (3)	0.83333 (11)	−0.33603 (10)	0.0274 (2)	
C13	0.25192 (4)	0.98089 (15)	−0.52385 (14)	0.0257 (3)	
H13	0.2577	1.0035	−0.6265	0.031*	
O1	0.10481 (3)	0.75465 (11)	−0.63146 (11)	0.0305 (2)	
N2	0.27422 (3)	1.03185 (13)	−0.41410 (12)	0.0281 (3)	
C7	0.12022 (4)	0.68615 (15)	−0.49215 (15)	0.0261 (3)	
H7	0.1238	0.5776	−0.5095	0.031*	
N1	0.17910 (3)	0.75114 (13)	−0.31966 (13)	0.0277 (3)	
C1	0.04117 (4)	0.75176 (18)	−0.10681 (16)	0.0330 (3)	
C4	0.09035 (4)	0.70947 (15)	−0.36207 (15)	0.0257 (3)	
C12	0.21739 (4)	0.88671 (15)	−0.48414 (14)	0.0246 (3)	
C8	0.16124 (4)	0.75648 (14)	−0.45511 (15)	0.0241 (3)	
C9	0.18390 (4)	0.83945 (15)	−0.56547 (14)	0.0248 (3)	
C3	0.08107 (4)	0.85489 (16)	−0.31074 (17)	0.0328 (3)	
H3A	0.0915	0.9380	−0.3630	0.039*	
C5	0.07352 (4)	0.58664 (15)	−0.28589 (16)	0.0288 (3)	
H5	0.0788	0.4894	−0.3212	0.035*	
C6	0.04879 (4)	0.60687 (17)	−0.15740 (17)	0.0331 (3)	
H6	0.0376	0.5242	−0.1066	0.040*	
C10	0.16899 (4)	0.85873 (17)	−0.72871 (15)	0.0317 (3)	
H10A	0.1909	0.8387	−0.8007	0.038*	
H10B	0.1595	0.9613	−0.7450	0.038*	
C11	0.13411 (5)	0.74717 (18)	−0.75476 (16)	0.0336 (3)	
H11A	0.1206	0.7696	−0.8524	0.040*	
H11B	0.1451	0.6455	−0.7609	0.040*	
C2	0.05646 (4)	0.87708 (17)	−0.18291 (18)	0.0353 (3)	
H2	0.0503	0.9741	−0.1490	0.042*	

### Atomic displacement parameters (Å^2^)

**Table:**

	*U*^11^	*U*^22^	*U*^33^	*U*^12^	*U*^13^	*U*^23^
Cl1	0.0505 (3)	0.0642 (3)	0.0388 (2)	0.0056 (2)	0.01860 (18)	−0.00215 (17)
O3	0.0336 (5)	0.0442 (6)	0.0257 (5)	−0.0137 (4)	0.0038 (4)	−0.0043 (4)
O2	0.0245 (4)	0.0339 (5)	0.0239 (5)	0.0006 (4)	0.0016 (3)	0.0031 (4)
C13	0.0275 (6)	0.0295 (7)	0.0201 (6)	0.0010 (5)	0.0023 (5)	−0.0011 (5)
O1	0.0294 (5)	0.0361 (5)	0.0260 (5)	−0.0023 (4)	−0.0017 (4)	0.0030 (4)
N2	0.0280 (5)	0.0322 (6)	0.0241 (5)	−0.0040 (5)	0.0045 (4)	−0.0013 (4)
C7	0.0275 (6)	0.0241 (6)	0.0268 (6)	−0.0003 (5)	0.0020 (5)	0.0010 (5)
N1	0.0245 (5)	0.0311 (6)	0.0277 (6)	0.0011 (4)	0.0031 (4)	0.0041 (4)
C1	0.0253 (6)	0.0453 (9)	0.0283 (7)	0.0026 (6)	0.0029 (5)	−0.0005 (6)
C4	0.0228 (6)	0.0265 (7)	0.0277 (7)	0.0003 (5)	0.0002 (5)	0.0010 (5)
C12	0.0276 (6)	0.0255 (6)	0.0207 (6)	0.0033 (5)	0.0025 (5)	−0.0009 (4)
C8	0.0253 (6)	0.0232 (6)	0.0238 (6)	0.0038 (5)	0.0022 (5)	0.0003 (4)
C9	0.0271 (6)	0.0235 (6)	0.0240 (6)	0.0006 (5)	0.0035 (5)	−0.0002 (5)
C3	0.0336 (7)	0.0261 (7)	0.0387 (8)	−0.0009 (6)	0.0061 (6)	0.0016 (5)
C5	0.0285 (6)	0.0251 (7)	0.0329 (7)	0.0009 (5)	0.0007 (5)	0.0027 (5)
C6	0.0313 (7)	0.0352 (8)	0.0327 (7)	−0.0025 (6)	0.0024 (5)	0.0081 (6)
C10	0.0363 (7)	0.0370 (8)	0.0219 (6)	−0.0085 (6)	−0.0013 (5)	0.0026 (5)
C11	0.0381 (8)	0.0406 (8)	0.0221 (7)	−0.0093 (6)	0.0007 (6)	−0.0014 (5)
C2	0.0344 (7)	0.0313 (8)	0.0401 (8)	0.0033 (6)	0.0040 (6)	−0.0053 (6)

### Geometric parameters (Å, °)

**Table:**

Cl1—C1	1.7456 (14)	C4—C5	1.3872 (18)
O3—N2	1.3934 (14)	C4—C3	1.3955 (19)
O3—H3	0.8200	C12—C9	1.3633 (18)
O2—C12	1.3651 (15)	C8—C9	1.4178 (17)
O2—N1	1.4105 (14)	C9—C10	1.4978 (18)
C13—N2	1.2742 (17)	C3—C2	1.386 (2)
C13—C12	1.4482 (18)	C3—H3A	0.9300
C13—H13	0.9300	C5—C6	1.3907 (19)
O1—C7	1.4351 (16)	C5—H5	0.9300
O1—C11	1.4409 (17)	C6—H6	0.9300
C7—C4	1.5099 (17)	C10—C11	1.525 (2)
C7—C8	1.5123 (18)	C10—H10A	0.9700
C7—H7	0.9800	C10—H10B	0.9700
N1—C8	1.3043 (18)	C11—H11A	0.9700
C1—C6	1.378 (2)	C11—H11B	0.9700
C1—C2	1.385 (2)	C2—H2	0.9300
			
N2—O3—H3	109.5	C12—C9—C10	134.78 (12)
C12—O2—N1	108.26 (9)	C8—C9—C10	121.57 (12)
N2—C13—C12	118.12 (11)	C2—C3—C4	120.87 (13)
N2—C13—H13	120.9	C2—C3—H3A	119.6
C12—C13—H13	120.9	C4—C3—H3A	119.6
C7—O1—C11	111.63 (10)	C4—C5—C6	120.92 (13)
C13—N2—O3	111.89 (10)	C4—C5—H5	119.5
O1—C7—C4	109.92 (11)	C6—C5—H5	119.5
O1—C7—C8	107.99 (10)	C1—C6—C5	118.75 (13)
C4—C7—C8	111.48 (11)	C1—C6—H6	120.6
O1—C7—H7	109.1	C5—C6—H6	120.6
C4—C7—H7	109.1	C9—C10—C11	107.63 (11)
C8—C7—H7	109.1	C9—C10—H10A	110.2
C8—N1—O2	105.46 (10)	C11—C10—H10A	110.2
C6—C1—C2	121.84 (13)	C9—C10—H10B	110.2
C6—C1—Cl1	118.81 (11)	C11—C10—H10B	110.2
C2—C1—Cl1	119.35 (12)	H10A—C10—H10B	108.5
C5—C4—C3	118.92 (12)	O1—C11—C10	111.33 (11)
C5—C4—C7	120.54 (12)	O1—C11—H11A	109.4
C3—C4—C7	120.44 (12)	C10—C11—H11A	109.4
C9—C12—O2	109.75 (11)	O1—C11—H11B	109.4
C9—C12—C13	132.92 (12)	C10—C11—H11B	109.4
O2—C12—C13	117.31 (11)	H11A—C11—H11B	108.0
N1—C8—C9	112.85 (12)	C1—C2—C3	118.65 (13)
N1—C8—C7	124.45 (11)	C1—C2—H2	120.7
C9—C8—C7	122.68 (12)	C3—C2—H2	120.7
C12—C9—C8	103.65 (11)		
			
C12—C13—N2—O3	179.32 (11)	O2—C12—C9—C10	−179.11 (14)
C11—O1—C7—C4	174.62 (11)	C13—C12—C9—C10	2.5 (3)
C11—O1—C7—C8	52.81 (13)	N1—C8—C9—C12	−0.76 (15)
C12—O2—N1—C8	0.96 (13)	C7—C8—C9—C12	177.73 (11)
O1—C7—C4—C5	121.01 (13)	N1—C8—C9—C10	179.62 (12)
C8—C7—C4—C5	−119.28 (13)	C7—C8—C9—C10	−1.90 (19)
O1—C7—C4—C3	−62.69 (16)	C5—C4—C3—C2	1.9 (2)
C8—C7—C4—C3	57.02 (16)	C7—C4—C3—C2	−174.40 (13)
N1—O2—C12—C9	−1.48 (14)	C3—C4—C5—C6	−2.0 (2)
N1—O2—C12—C13	177.20 (10)	C7—C4—C5—C6	174.35 (12)
N2—C13—C12—C9	165.24 (14)	C2—C1—C6—C5	1.9 (2)
N2—C13—C12—O2	−13.06 (18)	Cl1—C1—C6—C5	−177.63 (11)
O2—N1—C8—C9	−0.11 (14)	C4—C5—C6—C1	0.1 (2)
O2—N1—C8—C7	−178.57 (11)	C12—C9—C10—C11	167.22 (15)
O1—C7—C8—N1	161.63 (12)	C8—C9—C10—C11	−13.29 (18)
C4—C7—C8—N1	40.79 (17)	C7—O1—C11—C10	−73.78 (15)
O1—C7—C8—C9	−16.68 (16)	C9—C10—C11—O1	48.62 (16)
C4—C7—C8—C9	−137.52 (12)	C6—C1—C2—C3	−1.9 (2)
O2—C12—C9—C8	1.34 (14)	Cl1—C1—C2—C3	177.59 (11)
C13—C12—C9—C8	−177.05 (13)	C4—C3—C2—C1	0.0 (2)

### Hydrogen-bond geometry (°)

**Table:**

*D*—H···*A*
—···
